# Supplementary material for: Task complexity interacts with state-space uncertainty in the arbitration between model-based and model-free learning
Source: Nat Commun. 2019 Dec 16;10:5738. doi: 10.1038/s41467-019-13632-1 (PMC6915739; doi:10.1038/s41467-019-13632-1)
Supplement: Supplementary file 1 — Supplementary information [file 41467_2019_13632_MOESM1_ESM.pdf]

Supplementary Information

Task complexity interacts with state-space uncertainty in the arbitration between model-based and model-free learning.

Kim et al.

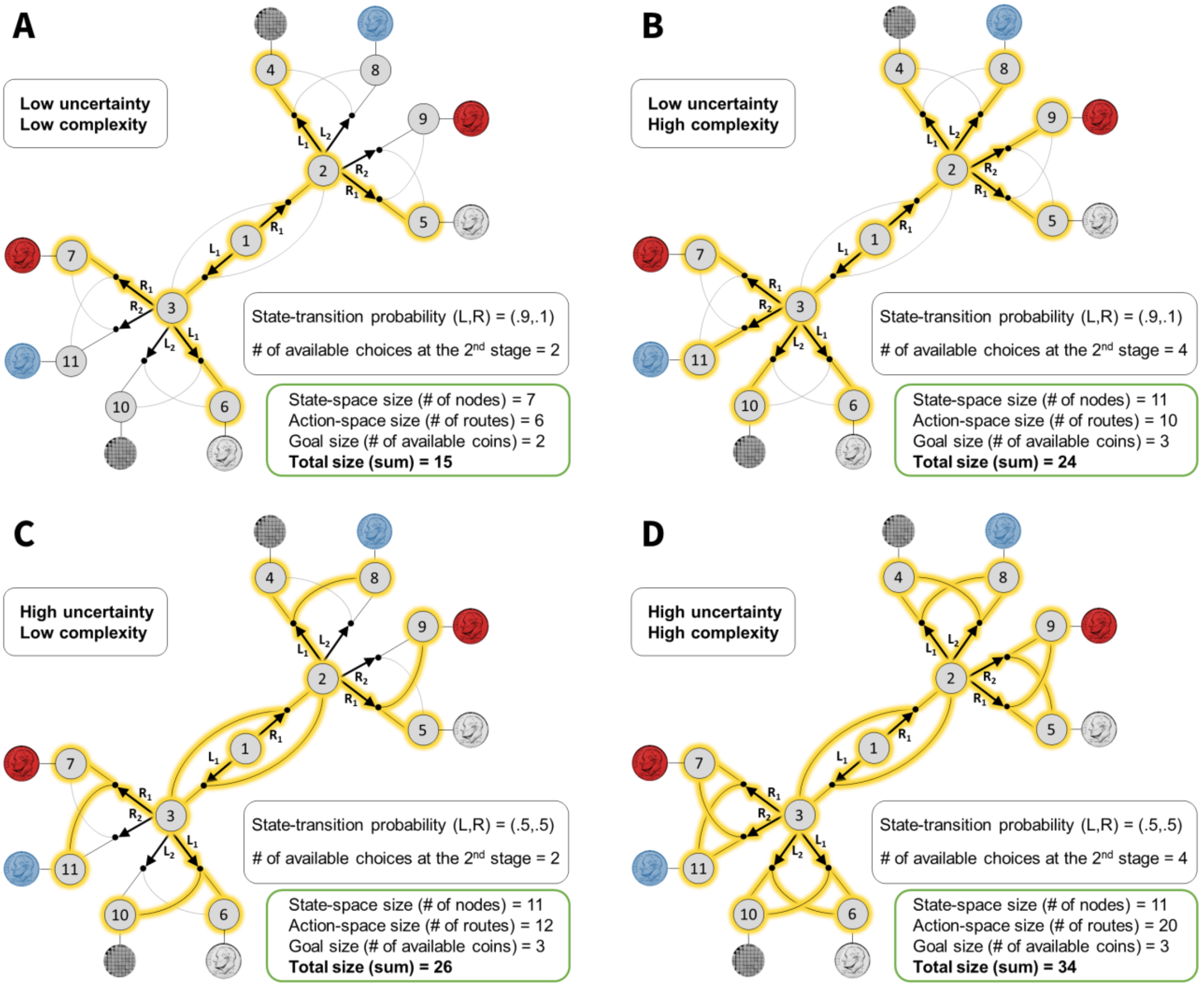

Supplementary Figure 1. Illustration of four different types of conditions in the task (low/high x uncertainty/complexity) Note that the association between goal types and coin types (color) were randomized for each subject. Shown in the rounded green box are the quantitative assessment of computational cost in each condition. Since there is no unique way of numerically coding or quantifying computational cost, our task design introduced three variables as a proxy for this: the size of state space, action space, and goal space, each of which can be numerically coded as the number of nodes, routes, and available coins, respectively. The estimated computational costs show that our task design can simulate conditions with different levels of computational load (the total computational load varying between 15 and 34).

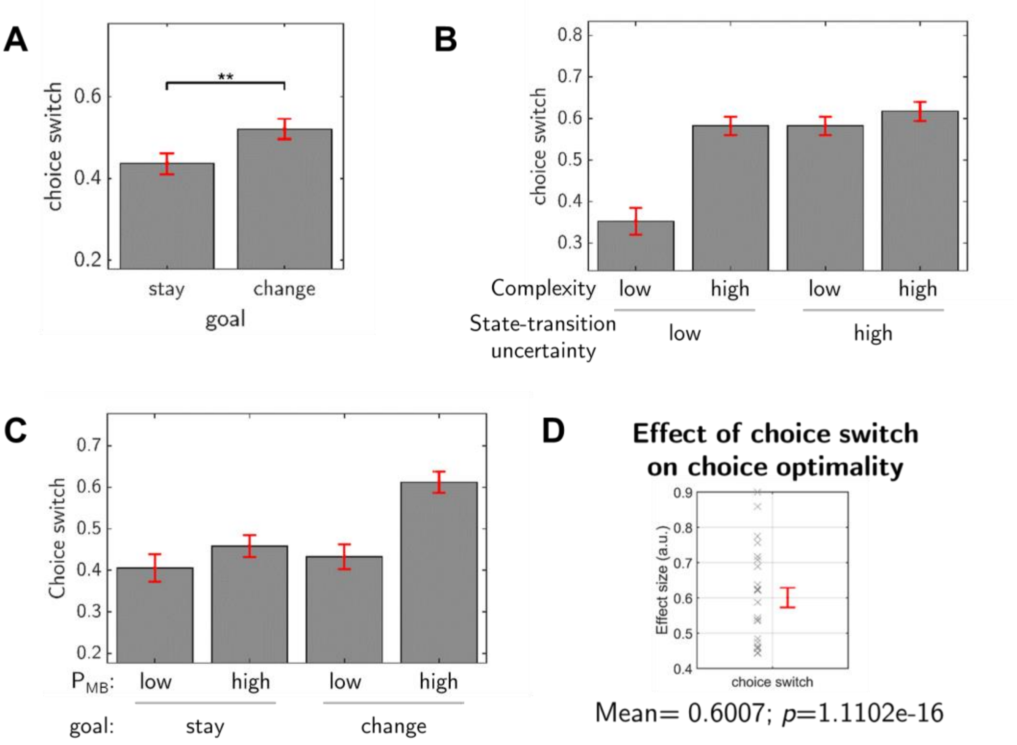

Supplementary Figure 2. Choice switching. (A) Choice switching is contingent on goal change. We examined choice behavior in a situation in which subjects need to set a new goal. A goal change necessitates a change in strategy, the degree to which people switch their strategy would relate to the extent to which they are engaging model-based control. Shown are subjects' ratio of choice switching in the second stage following goal change vs no goal change. (paired t-test; left p = 1.2e-4). Note that this measure is valid for only the second stage because in the first stage, each of the choices are optimal in half of the trials (in the high uncertainty condition). The goal-stay condition refers to the trials in which the *same* token values are being used on trial t and trial t+1 or at least token values even if different that would promote the same optimal choice on the proceeding compared to the next trial. The goal-change condition refers to the situation where the change in token values from trial t to trial t+1 necessitates a *change* in choice behavior from trial t to t+1. **(B)** Choice switching is sensitive to experimental conditions. To see if this choice behavior is affected by the experimental manipulation, we then examined the ratio of choice switching separately for each level of uncertainty and complexity. We found both a significant main and interaction effect of uncertainty and complexity on choice switching (two-way repeated measure ANOVA; p<0.001 for both main and interaction effects). The effect patterns are mostly consistent with that for choice optimality. Note however that the pattern of choice switching shown above does not completely align with choice optimality. This can be seen for the case of high uncertainty where choice switching frequency increases relative to the low uncertainty conditions,

whereas a decrease in choice optimality occurs in high uncertainty relative to low uncertainty conditions. This difference can be explained by the fact that switching choice can lead to subsequent choice of a good option or a poor option. To do the task effectively, one needs to switch to a good option (not merely increase switching rate per se). In the high uncertainty conditions we found that switching to the objectively better choice is indeed significantly reduced in this condition, compared to the low uncertainty conditions ($p<0.01$; paired t-test). We also found that subjects' earning ratio (= actual reward / maximum possible reward in each trial) is significantly reduced in the high uncertainty conditions relative to the other conditions ($p<0.001$; paired t-test). Taken together these findings provide an explanation for the behavioral underpinnings of the choice optimality measure reported in Figure 3C. **(C)** Link between choice switching and model-based control. We further investigated whether choice switching behavior can be diagnostic of model-based control. For this, we examined the ratio of choice switching is different as a function of the degree of model-based control (P_{MB} : the probability of choosing model-based strategy). We found both a significant main and interaction effect of model-based control (PMB) and goal change on choice switching (two-way repeated measure ANOVA; $p<0.001$ for the main effects and $p=0.001$ for the interaction effect). **(D)** Link between choice switching and choice optimality. Finally, in order to establish the relationship between choice switching and choice optimality, our behavioral measure indicating model-based control, we quantified the average correlation for each participant between choice optimality and the choice switching ratio. Specifically, we ran GLM analyses to compute an effect size for each individual subject, and found that all of the individual effect sizes are positive (mean effect size = 0.6; p=1.1e-16). We obtained the same result with the logistic regression analysis (mean effect size = 0.57; p=1.7e-8) Taken together, our findings help establish a link between the experimental manipulations (goal changes, uncertainty, and complexity), and the participants' choice behavior (choice switching), choice optimality, and learning strategy (model-based control). All error bars are SEM across subjects.

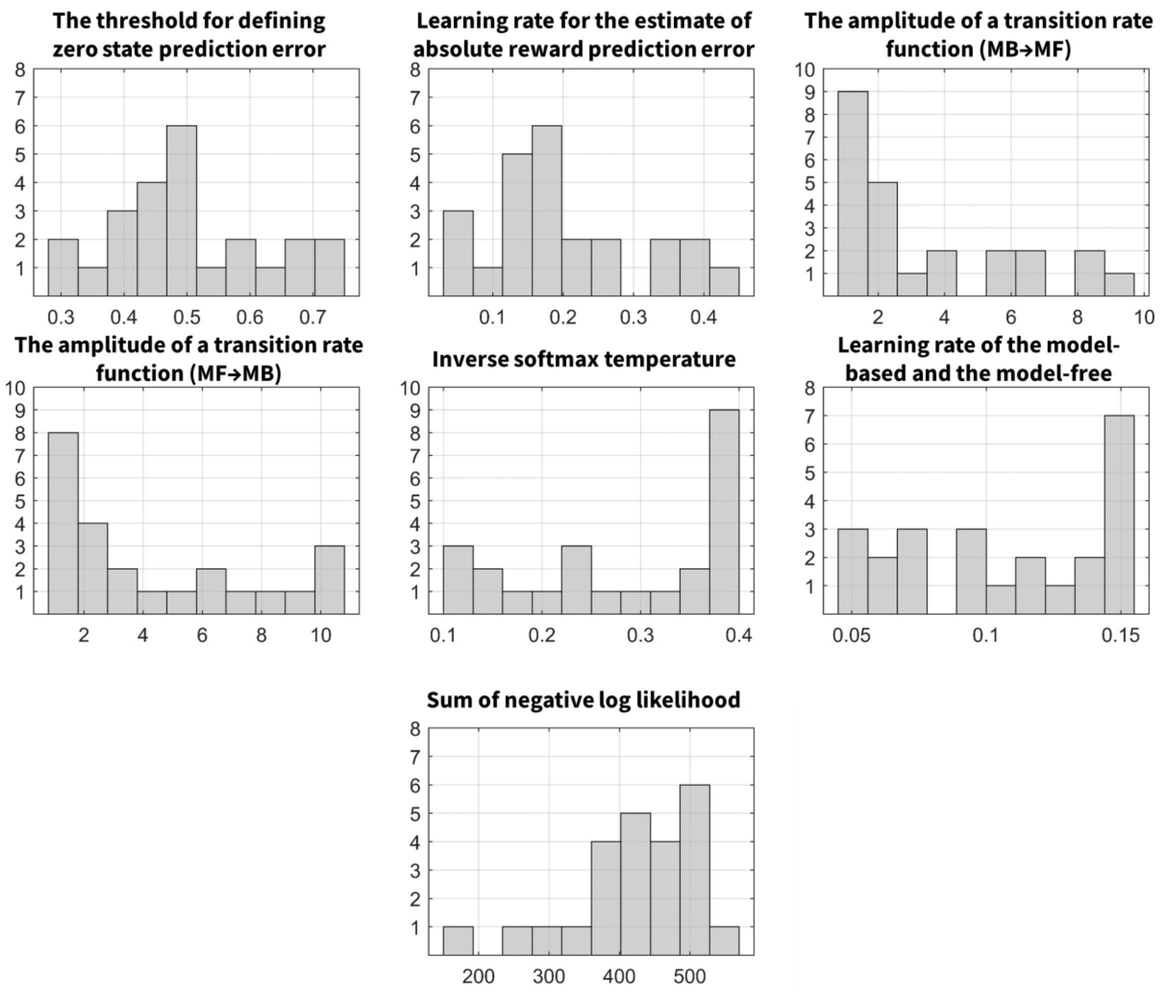

Supplementary Figure 3. Distributions of estimated parameter values of the model.

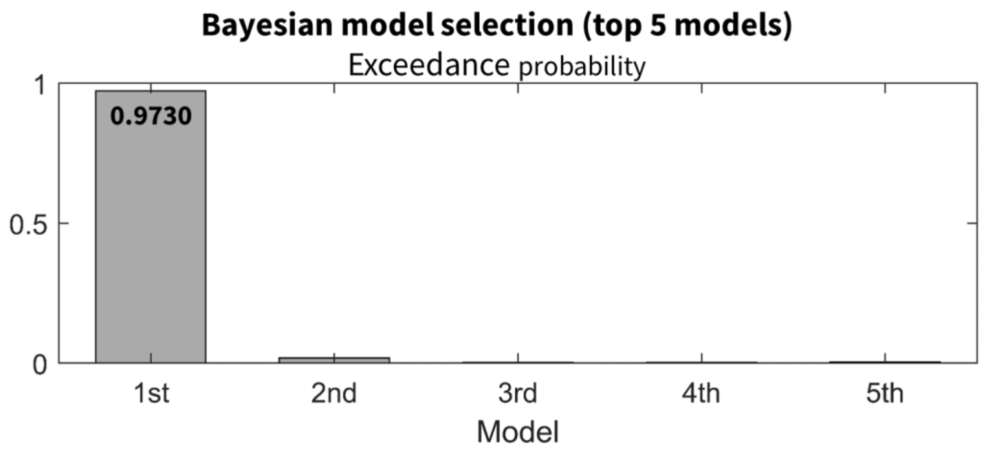

Details of the top 5 models

Model	Goal-driven MF type	Effect of complexity on transition between MB and MF RL			Effect of complexity on exploration
		Sign of modulation	Direction	Interaction	
1st	3Q	+	MF→MB	Interaction 2	Positive
2nd	3MF	+	MF→MB	Interaction 2	Positive
3rd	3MF	+	MF→MB	Interaction 2	Null
4th	1MF	+	MF→MB	Interaction 2	Null
5th	3MF	-	Bilateral	Interaction 2	Null

* Refer to Fig. 4 for more description about each hypothesis

Supplementary Figure 4. Bayesian model selection (BMS) on top 5 models, and computational hypotheses of those models.

Recoverability of the computational model's key parameters

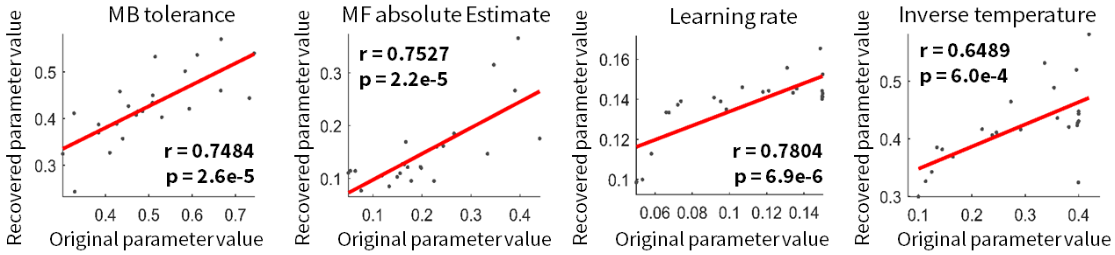

Supplementary Figure 5. Parameter recovery analysis. The parameter recovery analysis evaluates consistency between data-to-model parameter and model parameter-to-data conversion. The parameters from the best fitting model originally trained on actual subjects' data ("original parameter") were compared with the parameters from the models that were re-trained on simulated data ("recovered parameter"). The simulated data were generated by running simulations with the best fitting model on the original task.

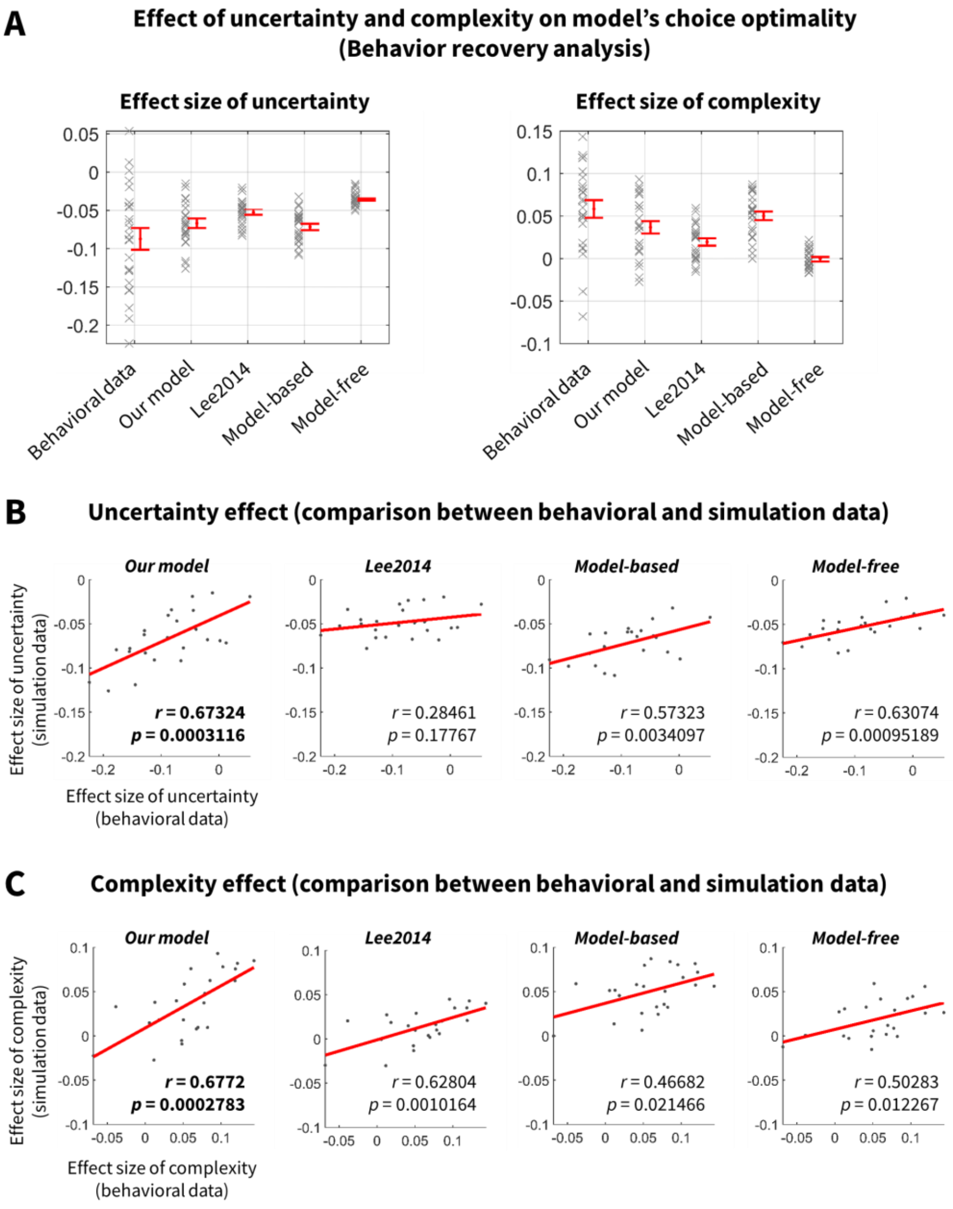

Supplementary Figure 6. Related to Figure 5. **(A)** The figures show the effect size of uncertainty and complexity on choice optimality of different models, including the best version of the model incorporating both uncertainty and complexity (Our model), the model incorporating uncertainty only (Lee2014), a pure model-based agent (Model-based), a pure model-free agent (Model-free). The effect sizes were computed by running a general linear model analysis with the choice optimality being included as a dependent variable, and uncertainty, complexity, reward values, choices in the previous trial, and goal values as independent variables (the same way as in Figure 5). The uncertainty and complexity, the two experimental variables of our task, are the two key factors that influences choice optimality (t-test; $p<0.001$). Error bars are SEM across subjects. **(B)**

Behavioral effect recovery analysis. The individual effect sizes of uncertainty/complexity on choice optimality of subjects (behavioral data) were compared with those of each model (simulated data).

Neural correlates of goal change

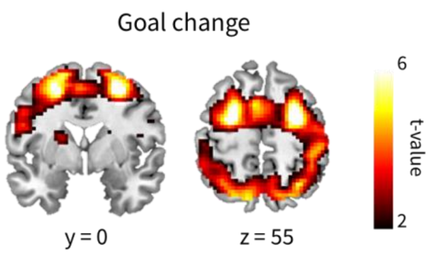

Supplementary Figure 7. Neural correlates of goal change. Medial frontal gyrus encodes a goal change signal indicating whether the goal needs to be changed from a previous trial. This signal is necessary for goal-driven MF RL. In all the brain images, statistical significancy of effects is illustrated by the heat colormap. Threshold set at $p<0.005$.

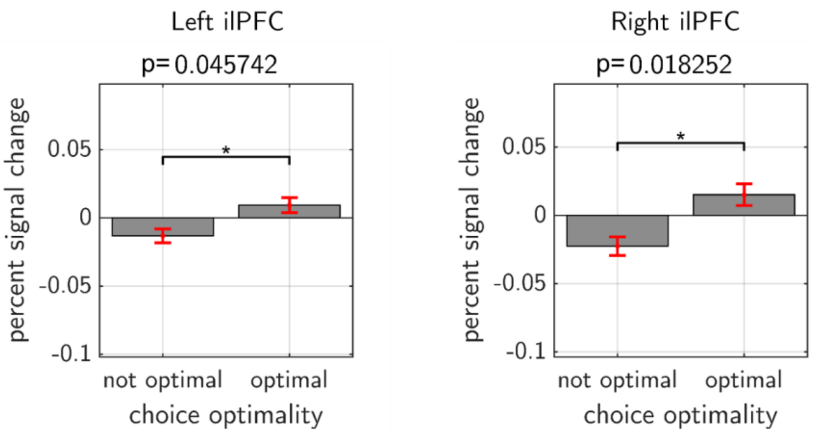

Supplementary Figure 8. Given that we have used choice optimality as a non-model-based index of model-based control (Figure 3), we have also now conducted an additional analysis in which we show evidence for choice optimality in the ilPFC ROI that we also identified as showing the computational signatures of the arbitration process. Consistent with the relationship between our computational model predictions, computational regressors found to be correlated with activity in the brain, and the relationship between choice optimality and model-based predictions, here we show that choice optimality is also reflected in our ROIs. Shown is a comparison between the trials in which choice optimality is high and low. This is consistent with the notion that when model-based control is increased, there is an increased activation in the vlPFC associated with an increased engagement of model-based control, and a decreased engagement of model-free control (the exact p-values for the contrast are shown above each plot). Error bars are SEM across subjects.

Supplementary Table 1. Estimated parameter values of the model (the best version according to the model comparison). Related to Fig. 4B.

Subject \ Parameter	1	2	3	4	5	6	Sum of negative log likelihood
1	0.3039	0.1476	1.1474	4.9426	0.3998	0.1467	320.2304
2	0.4099	0.3898	1.7325	7.2658	0.1251	0.0983	515.2891
3	0.3273	0.0641	1.0149	9.9897	0.2474	0.1500	369.7944
4	0.4531	0.3963	6.1229	1.6000	0.3540	0.0500	493.0697
5	0.6669	0.1782	8.5423	5.8020	0.1001	0.0501	518.4002
6	0.5085	0.1553	2.4221	1.6583	0.3363	0.0953	431.9479
7	0.5298	0.1970	8.1945	1.0182	0.2462	0.0675	565.2123
8	0.5934	0.1596	1.1625	4.2577	0.4000	0.0722	428.6207
9	0.5148	0.0760	6.6298	1.3031	0.3999	0.1500	176.0509
10	0.3015	0.2655	9.6187	1.0402	0.3979	0.0581	388.1172
11	0.3840	0.1493	1.0913	3.1196	0.3603	0.1211	403.4938
12	0.5837	0.1333	1.8027	6.7621	0.1137	0.1498	507.4127
13	0.3841	0.0540	1.0013	9.9992	0.2381	0.1497	464.7968
14	0.4858	0.1710	2.0061	2.7555	0.2732	0.1309	456.7124
15	0.6117	0.3467	3.8635	1.9669	0.3958	0.0533	423.1681
16	0.6662	0.1181	3.1976	1.2406	0.1345	0.1498	302.5826
17	0.7322	0.4402	1.0022	9.3621	0.3817	0.0739	485.2175
18	0.4333	0.1671	6.2510	1.2109	0.4000	0.1341	267.4762
19	0.4260	0.1986	1.8499	2.6964	0.2915	0.1179	441.3160
20	0.5091	0.2434	1.3119	1.9792	0.3996	0.0660	397.4218
21	0.4707	0.3337	5.4166	1.2503	0.1446	0.0917	509.1616
22	0.7432	0.0500	1.0114	8.0928	0.1650	0.1500	497.1235
23	0.4739	0.1642	1.0013	9.9274	0.3972	0.1361	385.1757
24	0.4397	0.2298	3.4788	3.4961	0.2193	0.1070	479.7771

Parameter: 1- the threshold for defining zero state prediction error, 2- learning rate for the estimate of absolute reward prediction error, 3- the amplitude of a transition rate function (MB→MF), 4- the amplitude of a transition rate function (MF→MB), 5- inverse softmax temperature, and 6- learning rate of the model- based and the model-free, respectively.

Supplementary Table 2. Neural signatures of the model-based, the model-free, and the arbitration system signals.

x	y	z	Peak in region	Hemi	p	# of voxels in the cluster	Z score	T score
		State prediction error (SPE)						
48	17	28	lPFC	R	0.000	73	5.62*	8.98
-36	14	28	lPFC	L	0.003	23	5.27*	7.92
33	20	1	Insula	R	0.013	8	4.97*	7.11
-30	17	1	Insula	L	0.069	-	2.76(1)	3.09
		Reward prediction error (RPE)						
-9	5	-8	Ventral striatum	L	0.010	-	3.52(2)	4.21
15	5	-8	Ventral striatum	R	0.046	-	2.42(3)	2.64
		Goal change						
-24	-4	52	MFG	R	0.009	13	4.98*	7.16
27	-1	55	MFG	L	0.013	14	4.89*	6.92
		Max reliability						
45	23	-11	ilPFC	R	0.000	197	4.55+	6.13
6	38	46	FPC	R	0.003	133	4.46+	5.94
-42	26	-2	ilPFC	L	0.021	84	4.53+	6.09
		Complexity (negative correlation)						
-18	5	58	SMA/MFG	L	0.039	98	3.58+	4.30
		Interaction of complexity and Max reliability – negative correlation						
54	23	7	ilPFC	R	0.003	157	4.75+	6.59
57	-40	4	STG	R	0.021	98	4.43+	5.88
-45	23	4	ilPFC	L	0.023	-	3.30(4)	3.86
		Chosen value of the goal-driven model-free (Q_{MF})						
-27	-1	61	SMA	L	0.000	123	5.80*	9.55
21	2	55	SMA	R	0.000	61	5.85*	9.73

-33	41	25	lPFC	L	0.002	31	5.34+	8.13
30	44	31	lPFC	R	0.000	225	4.68+	6.43
-36	-4	1	Posterior putamen	L	0.032	-	3.20(5)	3.71
Chosen value of the goal-driven model-free (Q_{MB})								
0	8	55	SMA	L/R	0.009	24	5.07*	7.37
-27	2	61	MFG	L	0.001	26	5.51*	8.63
27	8	49	MFG	R	0.016	6	4.93*	7.02
-30	53	13	lPFC	L	0.000	429	4.86+	6.86
Value difference of the arbitration system (chosen – unchosen)								
-12	23	-5	vmPFC	L	0.041	-	3.11(6)	3.57

* : threshold p < 0.05 FWE (voxel-level), the number of voxels in the cluster counted with the voxel-level threshold

+ : survives after whole-brain correction at the cluster-level (height threshold T = 3.55, threshold p < 0.05 FWE (cluster-level)), the number of voxels in the cluster counted with the cluster-level threshold

(1), (2), (3), (4), (5), (6) : survives after small-volume correction within the coordinate for each of the relevant contrasts from our original paper [1]. The number of voxels in the cluster is not indicated here since we are using voxel-based small-volume correction.

(1) : survives after small-volume correction within a 10-mm sphere centered coordinate (-30, 20, -2)

(2) : survives after small-volume correction within a 10-mm sphere centered coordinate (-9, 2, -8)

(3) : survives after small-volume correction within a 10-mm sphere centered coordinate (9, 5, -8)

(4) : survives after small-volume correction within a 10-mm sphere centered coordinate (-54, 38, 3)

(5) : survives after small-volume correction within a 10-mm sphere centered coordinate (-27, -4, 1)

(6) : survives after small-volume correction within a 10-mm sphere centered coordinate (-9, 29, -11)

lPFC : lateral prefrontal cortex, MFG : medial frontal gyrus, ilPFC : inferior lateral prefrontal cortex, FPC : frontopolar cortex, SMA : supplementary motor area, STG : superior temporal gyrus, vmPFC : ventromedial prefrontal cortex

Supplementary Table 3. Results of two-way repeated measures ANOVA. Related to Figure 3C.

Source	Uncertainty	Complexity	Type III sum of squares	Degree of freedom	Mean square	F	p
Uncertainty	Linear		.167	1	.187	38.655	.000
Error (Uncertainty)	Linear		.111	23	.005		
Complexity		Linear	.091	1	.091	31.546	.000
Error (Complexity)		Linear	.066	23	.003		
Uncertainty * Complexity	Linear	Linear	.047	1	.047	21.159	.000
Error (Uncertainty * Complexity)	Linear	Linear	.051	23	.002		

Supplementary Table 4. Results of two-way repeated measures ANOVA. Related to Figure 6C.

Source	Uncertainty	Complexity	Type III sum of squares	Degree of freedom	Mean square	F	p
Uncertainty	Linear		.008	1	.008	30.459	.000
Error (Uncertainty)	Linear		.006	23	.000		
Complexity		Linear	.109	1	.109	134.796	.000
Error (Complexity)		Linear	.019	23	.001		
Uncertainty * Complexity	Linear	Linear	.003	1	.003	4.803	.039
Error (Uncertainty * Complexity)	Linear	Linear	.013	23	.001		

Supplementary Methods

Behavioral measure (choice bias). In our task design, a left/right choice bias in the first stage can be interpreted as a behavioral marker indicating reward-based learning.

Our task design involves delicate manipulation of the goal. Note that the association between goal types and coin colors were randomized for each subject, and here we show one particular example (see the above figure). Let's define the R branch as the bottom left routes (accessible by making the R choice in the first stage) and the L branch as the top right routes (accessible by making the L choice in the first stage), respectively. The agent is not informed about task complexity until the second stage, so in the first stage a rational agent would make the following assumptions: in the first stage, outcome states associated with a silver coin are accessible by making a primary choice in both branches. An outcome state associated with a red coin are accessible by making a primary and a secondary choice in the R and L branch, respectively. An outcome state associated with a blue coin are accessible by making a secondary choice in both the R and the L branch.

Accommodating this situation, we can roughly calculate the expected value of the L/R choice of the optimal agent (the same agent used to compute choice optimality) for the first stage. The probability of transitioning to a desired outcome state by making a primary and a secondary choice is given by (0.7, 0.3), which is computed by taking average of the two state-transition probability values: (0.9, 0.1) and (0.5, 0.5). Note that this setting is used to simulate average experimental conditions. For the sake of simplicity, reward values were normalized to 1 (for the goal coin), 0.5 (for the other coins), and 0 (unrewarded).

If we assume that an agent relies on model-free control and that the agent makes a greedy choice, we can compute the expected values and the corresponding choice biases by using the uniform state-transition probability distribution (0.5,0.5) (meaning that the agent is agnostic about state-transition uncertainty and thus cannot afford to accommodate state-transition probability value changes) as follows. Note that the low, medium, and high value coin corresponds to silver, red, and blue coins in Figure 1 and Supplementary Figure 1; again, the coin colors are randomized for each subject.

- Silver coin : (the expected value of L branch) 1x0.5 + 0x0.5 = 0.5. (the expected value of R branch) 1x0.5 + 0.5x0.5 = 0.75. The expected value difference (L-R) = -0.25. We expect a R choice bias.

- Red coin : (the expected value of L branch) 1x0.5 + 0.5x0.5 = 0.75. (the expected value of R branch) 0.5x0.5 + 1x0.5 = 0.75. Therefore expected value difference (L-R) = 0. We expect no L choice bias.

- Blue coin : (the expected value of L branch) 0.5x0.5 + 1x0.5 = 0.75. (the expected value of R branch) 0x0.5 + 1x0.5 = 0.5. Therefore expected value difference (L-R) = +0.25. We expect L choice bias.

Therefore, if subjects performed the task using pure model-free control, they would show a well-balanced choice bias pattern: R bias, zero bias, and L bias for each goal, respectively.

On the other hand, if we assume that an agent relies on model-based control, we can compute the expected values and the corresponding choice biases, this time by using the average state-transition probability set (0.7,0.3) (meaning that the agent actively accommodates state-transition probability value changes between (0,9,0.1) and (0.5,0.5)) as follows.

- Silver coin : (the expected value of L branch) 1x0.7 + 0x0.3 = 0.7. (the expected value of R branch) 1x0.7 + 0.5x0.3 = 0.85. The expected value difference (L-R) = -0.15. We expect a weak R choice bias.

- Red coin : (the expected value of L branch) 1x0.7 + 0.5x0.3 = 0.85. (the expected value of R branch) 0.5x0.7 + 1x0.3 = 0.65. Therefore expected value difference (L-R) = +0.2. We expect a weak L choice bias.

- Blue coin : (the expected value of L branch) 0.5x0.7 + 1x0.3 = 0.65. (the expected value of R branch) 0x0.7 + 1x0.3 = 0.3. Therefore expected value difference (L-R) = +0.35. We expect L choice bias.

Therefore, if subjects performed the task using model-based control, they would exhibit a slight left bias pattern: weak R bias, weak L bias, and L bias for each goal, respectively.

Behavioral measure (choice optimality). Choice consistency, a conventional behavioral measure used to quantify insensitivity to changes in the environmental structure (one of the key characteristics of model-free RL), works well for conventional two-step task paradigms in which the environment is stable for a certain period [2,3]. Unfortunately, Choice consistency is not a suitable measure for our highly dynamic task design in which we manipulate task complexity for the following reasons: First, reward values fluctuate on a trial-by-trial basis. This manipulation encourages trial-by-trial arbitration between model-free and model-based control. Choice behavior on each trial is affected by the relative values of each coins, nullifying the choice

consistency effect. Second, the level of state-space complexity also varies on a trial-by-trial basis. State-space complexity is manipulated by varying the number of available choices. The choice consistency rate would plummet when the number of available choices increases from 2 to 4. Third, the independent manipulation of the first two factors (state-space complexity and reward value) further promotes arbitration. For example, if the values of the three coins remain constant on each trial, then it's likely that choice behavior would converge to a specific sequence of choices for each task complexity condition, which does not necessitate arbitration control. Fourth, most of the goals are achievable in state1 regardless of experimental conditions. This means that there are usually more than two different behavioral policies or pathways/outcome states that enable a subject to achieve a goal (coin). These factors make it difficult to apply choice consistency to the present task design.

To deal with all the above issues, we devised an alternative behavioral measure that is robust against the above-mentioned experimental issues: choice optimality. This measure quantifies the extent to which participants on a given trial took the objectively best choice had they complete access to the task state-space, and a perfect ability to plan actions in that state-space. It is based on the choice of the ideal agent assumed to have a full, immediate access to information of the environmental structure, including state-transition uncertainty and task complexity. The choice optimality is defined as the degree of match between subjects' actual choices and an ideal agent's choice corrected for the number of available options. To compute the degree of choice match between the subject and the ideal agent, for each condition, we calculated an average of normalized values (i.e., likelihood) of the ideal agent for the choice that a subject actually made on each trial. To correct for the number of options, we then multiplied it by 2 for the high complexity condition; this is intended to compensate for the effect that the baseline level of the likelihood in the high complexity condition (# of available options =4) becomes half of that in the low complexity condition (# of available options =2). In other words, this adjustment effectively compensates the effect of # of available options on normalization without biasing the correspondence between participant's choices and optimal choices. The choice optimality value would have a maximum/minimum value if a subject made the same/opposite choice as the ideal agent's in all trials, regardless of complexity condition changes.

Owing to the fact that the ideal agent's behavioral policy is not affected by the variability of such experimental variables, this measure serves as a reasonable proxy for assessing the degree of participants' engagement in model-based control. In principle, provided that the model-based

agent has complete knowledge of the state-space and no cognitive constraints, it will always choose more optimally than a model-free agent.

Reference

1. Lee, S. W., Shimojo, S. & O'Doherty, J. P. Neural Computations Underlying Arbitration between Model-Based and Model-free Learning. *Neuron* **81**, 687–699 (2014).
2. Daw, N. D., Gershman, S. J., Seymour, B., Dayan, P. & Dolan, R. J. Model-based influences on humans' choices and striatal prediction errors. *Neuron* **69**, 1204–15 (2011).
3. Miller, K. J., Botvinick, M. M. & Brody, C. D. Dorsal hippocampus contributes to model-based planning. *Nat. Neurosci.* **20**, 1269–1276 (2017).
