# Supplementary material for: Task complexity interacts with state-space uncertainty in the arbitration between model-based and model-free learning
Source: Nat Commun. 2019 Dec 16;10:5738. doi: 10.1038/s41467-019-13632-1 (PMC6915739; doi:10.1038/s41467-019-13632-1)
Supplement: Supplementary file 3 — Reporting Summary [file 41467_2019_13632_MOESM3_ESM.pdf]

nature research

Corresponding author(s): John O'Doherty and Sang Wan Lee

Last updated by author(s): Oct 29, 2019

Reporting Summary

Nature Research wishes to improve the reproducibility of the work that we publish. This form provides structure for consistency and transparency in reporting. For further information on Nature Research policies, see Authors & Referees and the Editorial Policy Checklist.

Statistics

For all statistical analyses, confirm that the following items are present in the figure legend, table legend, main text, or Methods section.

n/a	Confirmed	
☐	☒	The exact sample size (n) for each experimental group/condition, given as a discrete number and unit of measurement
☐	☒	A statement on whether measurements were taken from distinct samples or whether the same sample was measured repeatedly
☐	☒	The statistical test(s) used AND whether they are one- or two-sided<br>*Only common tests should be described solely by name; describe more complex techniques in the Methods section.*
☐	☒	A description of all covariates tested
☐	☒	A description of any assumptions or corrections, such as tests of normality and adjustment for multiple comparisons
☐	☒	A full description of the statistical parameters including central tendency (e.g. means) or other basic estimates (e.g. regression coefficient) AND variation (e.g. standard deviation) or associated estimates of uncertainty (e.g. confidence intervals)
☐	☒	For null hypothesis testing, the test statistic (e.g. F, t, r) with confidence intervals, effect sizes, degrees of freedom and P value noted<br>*Give P values as exact values whenever suitable.*
☒	☐	For Bayesian analysis, information on the choice of priors and Markov chain Monte Carlo settings
☒	☐	For hierarchical and complex designs, identification of the appropriate level for tests and full reporting of outcomes
☐	☒	Estimates of effect sizes (e.g. Cohen's d, Pearson's r), indicating how they were calculated

Our web collection on statistics for biologists contains articles on many of the points above.

Software and code

Policy information about availability of computer code

Data collection	Functional imaging was performed on a 3T Siemens (Erlangen, Germany) Tim Trio scanner located at the Caltech Brain Imaging Center (Pasadena, CA) with a 32 channel radio frequency coil for all the MR scanning sessions. To reduce the possibility of head movement related artifact, participants' heads were securely positioned with foam position pillows. High resolution structural images were collected using a standard MPRAGE pulse sequence, providing full brain coverage at a resolution of 1 mm × 1 mm × 1 mm. Functional images were collected at an angle of 30° from the anterior commissure-posterior commissure (AC-PC) axis, which reduced signal dropout in the orbitofrontal cortex. Forty-five slices were acquired at a resolution of 3 mm × 3 mm × 3 mm, providing whole-brain coverage. A one-shot echo-planar imaging (EPI) pulse sequence was used (TR = 2800 ms, TE = 30 ms, FOV = 100 mm, flip angle = 80°).
Data analysis	The SPM12 software package was used to analyze the fMRI data (Wellcome Department of Imaging Neuroscience, Institute of Neurology, London, UK). The first four volumes of images were discarded to avoid T1 equilibrium effects. Slice-timing correction was applied to the functional images to adjust for the fact that different slices within each image were acquired at slightly different points in time. Images were corrected for participant motion, spatially transformed to match a standard echo-planar imaging template brain, and smoothed using a 3D Gaussian kernel (6 mm FWHM) to account for anatomical differences between participants. This set of data was then analyzed statistically. A high-pass filter with a cutoff at 129 seconds was used

For manuscripts utilizing custom algorithms or software that are central to the research but not yet described in published literature, software must be made available to editors/reviewers. We strongly encourage code deposition in a community repository (e.g. GitHub). See the Nature Research guidelines for submitting code & software for further information.

Data

Policy information about availability of data

All manuscripts must include a data availability statement. This statement should provide the following information, where applicable:

- Accession codes, unique identifiers, or web links for publicly available datasets
- A list of figures that have associated raw data
- A description of any restrictions on data availability

The raw behavioral data, simulation codes, and fMRI results are available for download at https://github.com/brain-machine-intelligence/task_complexity_2018 .

Field-specific reporting

Please select the one below that is the best fit for your research. If you are not sure, read the appropriate sections before making your selection.

☒ Life sciences ☐ Behavioural & social sciences ☐ Ecological, evolutionary & environmental sciences

For a reference copy of the document with all sections, see nature.com/documents/nr-reporting-summary-flat.pdf

Life sciences study design

All studies must disclose on these points even when the disclosure is negative.

Sample size	n=24 for behavioral analysis, and n=22 for fMRI analysis.
Data exclusions	Subjects were screened prior to the experiment to exclude those with a history of neurological or psychiatric illness.
Replication	All attempts at replication were successful.
Randomization	There was no allocation into any experimental group in the paper.
Blinding	There was no allocation into any experimental group in the paper.

Reporting for specific materials, systems and methods

We require information from authors about some types of materials, experimental systems and methods used in many studies. Here, indicate whether each material, system or method listed is relevant to your study. If you are not sure if a list item applies to your research, read the appropriate section before selecting a response.

Materials & experimental systems

n/a	Involved in the study	
☒	☐	Antibodies
☒	☐	Eukaryotic cell lines
☒	☐	Palaeontology
☒	☐	Animals and other organisms
☐	☒	Human research participants
☒	☐	Clinical data

Methods

n/a	Involved in the study	
☒	☐	ChIP-seq
☒	☐	Flow cytometry
☐	☒	MRI-based neuroimaging

Human research participants

Policy information about studies involving human research participants

Population characteristics	Twenty four right-handed volunteers (ten females, with an age range between 19 and 55) participated in the study, 22 of whom were scanned with fMRI.
Recruitment	Subjects were screened prior to the experiment to exclude those with a history of neurological or psychiatric illness.
Ethics oversight	All participants gave informed consent, and the study was approved by the Institutional Review Board of the California Institute of Technology.

Note that full information on the approval of the study protocol must also be provided in the manuscript.

Magnetic resonance imaging

Experimental design

Design type	Event-related
Design specifications	In the pre-training session, subjects were given 100 trials in which they can freely navigate the state space by making any choices. The experiment proceeded in five separate scanning sessions of 80 trials each on average.
Behavioral performance measures	As a result of the task, participants receive points.

Acquisition

Imaging type(s)	functional
Field strength	3T
Sequence & imaging parameters	A one-shot echo-planar imaging (EPI) pulse sequence was used (TR = 2800 ms, TE = 30 ms, FOV = 100 mm, flip angle = 80°).
Area of acquisition	a whole brain

Diffusion MRI ☐ Used ☒ Not used

Preprocessing

Preprocessing software	SPM12
Normalization	SPM adopts both linear and non-linear transformation. Linear transformation is affine transformation fits using 12 parameters (3 translations, 3rotations, 3 shears, and 3 zooms). Non-linear registration warps images to fit to a standard template (MNI).
Normalization template	MNI
Noise and artifact removal	High frequency cut-off filter applied.
Volume censoring	SPM12

Statistical modeling & inference

Model type and settings	Multivariate general linear modeling (GLM); random-effect
Effect(s) tested	Regressors extracted from computational model, and parametric effects of the regressors are tested.

Specify type of analysis: ☐ Whole brain ☐ ROI-based ☒ Both

Anatomical location(s)	Right ilPFC (45, 23, -11), left ilPFC (-42, 26, -2) and (-54, 38, 3), posterior putamen (-27,-4,1), ventromedial prefrontal cortex (-9,29,-11), ventral striatum (-9,2,-8) and (9,5,-8)
Statistic type for inference (See Eklund et al. 2016)	Voxel-wise, cluster-level, and small-volume correction were used.
Correction	FWE corrected p value.

Models & analysis

n/a	Involved in the study
☒	☐ Functional and/or effective connectivity
☒	☐ Graph analysis
☐	☒ Multivariate modeling or predictive analysis

Multivariate modeling and predictive analysis	We created subject-specific design matrices containing the following regressors: (1) state prediction error, (2) reward prediction error, (3) a parametric regressor encoding the goal change, (4) maximum reliability of MB and MF, (5) the action value of MB (Q_MB), (6) the action value of MF (Q_MF), (7) a weighted sum of the (5) and (6), according to the output of the arbitration system (Q_ARB).
